# Capillary flow homogenization during functional activation revealed by optical coherence tomography angiography based capillary velocimetry

**DOI:** 10.1038/s41598-018-22513-4

**Published:** 2018-03-07

**Authors:** Yuandong Li, Wei Wei, Ruikang K. Wang

**Affiliations:** Department of Bioengineering, University of Washington, Seattle, USA

## Abstract

Elaborate modeling study suggests an important role of capillary transit time heterogeneity (CTTH) reduction in brain oxygenation during functional hyperemia. Here, we use optical coherence tomography angiography (OCTA) capillary velocimetry to probe blood flow dynamics in cerebral capillary beds and validate the change in CTTH during functional activation in an *in vivo* rodent model. Through evaluating flow dynamics and consequent transit time parameters from thousands of capillary vessels within three-dimensional (3-D) tissue volume upon hindpaw electrical stimulation, we observe reductions in both capillary mean transit time (MTT) (9.8% ± 2.2) and CTTH (5.9% ± 1.4) in the hindlimb somatosensory cortex (HLS1). Additionally, capillary flow pattern modification is observed with a significant difference (*p* < 0.05) between the HLS1 and non-activated cortex regions. These quantitative findings reveal a localized microcirculatory adjustment during functional activation, consistent with previous studies, and support the critical contribution of capillary flow homogenization to brain oxygenation. The OCTA velocimetry is a useful tool to image microcirculatory dynamics *in vivo* using animal models, enabling a more comprehensive understanding as to hemodynamic-metabolic coupling.

## Introduction

Normal brain function depends on the regulation of oxygen supply through bloodstream to support the actively changing metabolic needs^1^. The temporal and spatial relationship between neuronal activity and cerebral blood flow (CBF), termed neurovascular coupling^2^, has been utilized and studied in functional magnetic resonance imaging (fMRI)^3–5^. Specifically, the hemodynamic response during neurovascular coupling has been observed in fMRI as localized increase of CBF exceeding that of cerebral metabolic rate of oxygen (CMRO_2_)^6^, giving rise to a lower deoxyhemoglobin concentration in brain tissue and hence blood oxygen level dependent (BOLD) signal contrast for functional brain mapping^7–9^. Despite decades of effort in analyzing the functional relationship between CBF and brain oxygenation, our understanding of such flow-metabolism coupling remains incomplete. The disproportionate elevation of CBF in comparison to the relatively increased CMRO_2_^10,11^, particularly, suggests an involvement of additional factors in the non-linear coupling between oxygen consumption and the extent of hyperemia^12^.

**Figure 1:**
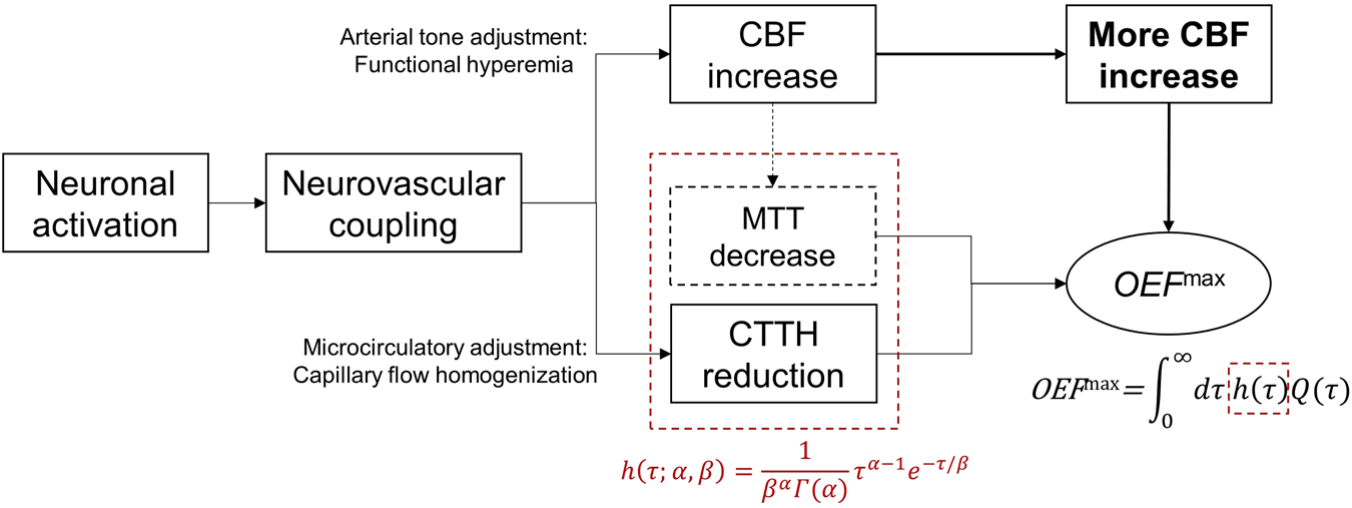
Schematic overview of the Jespersen&Østergaard^13^ model of CTTH effect on brain oxygenation. The upper thread refers to arterial tone adjustment in neurovascular coupling during functional activation, representing as functional hyperemia in observation with excessive increase of CBF (relative to the increase of CMRO^2^). The lower thread represents microcirculatory adjustment during functional activation involving capillary flow homogenization. In this work, capillary flow dynamics are modeled with capillary transit time *τ* distribution in gamma function *h*(*τ*), where MTT is determined by the mean *αβ* of the gamma function and CTTH is determined by the standard deviation $\sqrt{\alpha }\beta$. The OEF of the entire capillary bed is calculated from a single capillary contribution *Q*(*τ*) weighted by distribution *h*(*τ*). During cortical activation, assuming *CBV’* = *CBF·MTT* is constant according to the central volume theorem, the inherent reduction of OEF due to decreased MTT must be accompanied by CTTH reduction (capillary homogenization) in order to secure adequate level of oxygenation to achieve *OEF* ^max^ during functional hyperemia.

Recently, Jespersen & Østergaard^13^ have revisited the flow-diffusion function of oxygen, taking into account of the heterogeneous distribution of the red blood cells (RBC) transit times across capillary bed, to evaluate oxygen extraction in cerebral tissue. Accordingly, they have modeled the combined effects of CBF and capillary transit time heterogeneity (CTTH) on the maximum oxygen extraction fraction (*OEF*^max^). Briefly in this elegant theoretical model^13^ (Fig. 1), the hemodynamic contribution to *OEF*^max^ is determined by both vascular mean transit time (MTT), which is inversely related to CBF according to central volume theorem^14^, and CTTH, which is quantified as the standard deviation of the RBC transit time distribution across capillary bed. During functional activation, the inherent reduction of OEF due to the initial CBF increase is counteracted by capillary transit time homogenization, hence CTTH reduction, which would secure sufficient oxygenation during the subsequent (or simultaneous) episodes of hyperemia to meet the increased metabolic demand of oxygen in the activated tissue bed. This model has provided biophysical support to the disproportionate increase in CBF seen in functional activation, and established a framework for the quantitative characterization of capillary flow adjustment in neurovascular coupling.

The *in vivo* imaging of capillary flow dynamics and the validation of CTTH hypothesis have challenged the current microscopic neuroimaging techniques. Bolus tracking techniques with high resolution two-photon microscopy (TPM)^15^ or confocal laser scanning fluorescence microscopy^16^ have been previously used to estimate MTT and CTTH in rodent brains, based on measurements of a bolus plasma dye passage through cortical vasculature. Due to the incapability of direct imaging of RBC in single capillary vessels, however, spatial distribution of RBC transit times at capillary bed was unobtainable for an accurate CTTH evaluation^17^. Alternatively, single-line scanning velocimetry using TPM measures RBC speed directly in individual capillary passages^18^. Despite of a promising results of capillary velocity and flux quantification using line-scanning velocimetry in steady-state brain^19^, the technique is limited in its data acquisition speed (hours)^20^, as well as in an inadequate sampling size (<100 capillaries)^19^ for CTTH assessment with sufficient statistical power. Optical coherence tomography angiography (OCTA) has enabled three-dimension (3-D) quantitative imaging of blood flow in cerebral arteries and veins^21^, as well as visualization of the microvasculature at capillary level by analyzing the dynamic scattering signals embedded within the tissue volume^22^. As OCTA obtains flow information over a generally large focal depth^20^ in cerebral tissue together with micron-scale resolution^23^, the limitations in line-scanning TPM can be potentially lifted by imaging a larger amount (hundreds to thousands) of capillaries in 3-D space within tens of seconds^24^. Nevertheless, the accurate characterization of capillary flow speeds with sufficiently high statistical power remains a challenge for the use of current OCTA technique to investigate hemodynamic responses during cortical activities. In a recent study, Lee *et al*. have developed and applied statistical intensity variation analysis with OCTA for tracing changes in RBC flux over hundreds of capillaries within ~1 s^25^. Using such high-throughput monitoring of RBC flux dynamics, they have revealed capillary flux homogenization in rat somatosensory cortex during forepaw electrical stimulation, which showed the potential OCTA based techniques to study microcirculatory dynamics during neural activation.

Recently, based on OCTA, we have developed a statistical method of eigen-decomposition (ED) analysis^26^ to extract the frequency components of dynamic capillary flow from the coherent optical signals generated in high-speed OCT scans. The preliminary results have indicated a linear relationship between the measured mean frequency (MF) and the mean RBC velocity in individual capillary passages^26^. The method has been successfully applied to quantify capillary flow parameters in mice by measuring thousands of capillaries in one volumetric dataset with 50 μs temporal resolution, which revealed MTT and CTTH differences in mouse somatosensory cortex before and after ischemic stroke insult^26^.

In this paper, we applied this novel approach of ED analysis with OCTA to study the microcirculatory adjustment during stimulus-evoked cortical activation. Guided with the oxygenation mapping using laser speckle contrast imaging (LSCI)^27^, we performed OCTA velocimetry scans at both activated and non-activated cortex based on oxygen consumption, and correlated, for the first time, capillary flow responses to oxygen metabolism signal. We aimed to utilize the statistical powered OCTA results to validate, *in vivo*, the local CTTH reduction during functional hyperemia proposed in previous modeling study.

## Material and Methods

### Animal preparation

All animal experimental procedures in this study were approved by the Institutional Animal Care and Use Committee (IACUC) of the University of Washington and conduced in accordance with University of Washington guidelines and ARRIVE guidelines.

C57BL/6 mice (Charles River Laboratories, n = 12, 3-month-old, 23–25 g) were used under isoflurane anesthesia with a mixture of 0.2 L/min pure oxygen and 0.8 L/min air. Physiological parameters were monitored, including adequate anesthesia depth (no hindpaw reflexes), blood pressure, heart rate, and body temperature (36.8 ± 0.2 °C) throughout all experimental procedures. Cranial window procedures were conducted similar to that described previously by Li *et al*.^28^.

### Hindpaw electrical stimulation and laser speckle contrast imaging (LSCI)

Two 30-gauge needles inserted into the plantar surface of the mouse hindpaw, contralateral body side to cranial window, were connected to +/− outputs of a square pulse stimulator (SD9, Grass Instruments Medical) to deliver square wave voltage pulses^24^. A 4.1kΩ resistor was connected between the return electrode needle and the machine output to identify the stimulus current, which was calculated with a peak voltage applied to the resistor detected on a digital oscilloscope. Each trial of stimulation consists of 20 min resting time before 30 s stimulation with an amplitude of 2 mA in 0.3 ms duration repeated at 3 Hz. Each animal received 3 trials of electrical stimulation. The first trial was performed under LSCI imaging to localize oxygenation signal at HLS1 during stimulation. Briefly in this imaging method, a differential model^27^, based on the difference in absorption between two wavelengths, was used to estimate the changes in oxy- (ΔHbO) or deoxyhemoglobin (ΔHb), and the concentration changes of Hb was mapped where Hb of 40 μM in the resting brain was assumed^29^. The other two trials of stimulation were performed for OCTA velocimetry scans, one at HLS1 region, and the other at a non-activated control region (CTRL) identified from LSCI oxygenation mapping.

### OCTA imaging

OCT angiograms and capillary velocity mapping were obtained using an in-house spectral-domain OCT (SD-OCT) system^28,30^. Briefly, the system was equipped with a broadband super-luminescent diode (SLD) light source (LS2000B, Thorlabs Inc.) with a center wavelength of 1340 nm and a spectral bandwidth of 110 nm, giving an axial resolution of ~6 µm in tissue. A 10× objective lens was used to focus the light into the brain subsurface cortex of the animal, providing a lateral resolution of ~10 µm. Details of the system configuration can be found in ref.^30^.

Typical cerebral angiogram within the cranial window (4 × 4 mm) was first produced in resting-state brain using traditional optical microangiography (OMAG) protocol^31^, where 400 A-lines in the depth axis (z) was acquired within each B-scan at A-line rate of 92 kHz, and 8 repeated B-scans were performed at each of the 400 cross-sectional locations (z-x).

OCTA capillary velocimetry was then performed at HLS1 and CTRL cortex by simply targeting the designated cortex regions identified by the LSCI oxygenation maps. Prior to stimulation, capillary velocimetry scans at two regions were acquired during resting-state, then scans were performed during trials of electrical stimulation. The OCTA velocimetry scanning protocol^26^ consists of 50 repeated A-lines at each spatial position, i.e. M-scan, at a rate of 20 kHz (time interval 50 µs between successive scans). A total of 200 positions within each B-scan (x) and 100 spatial locations (i.e. B-scans) in the slow axis (y) were completed within 75 s, covering a region of 1.5 × 0.75 mm (x-y) with a uniform transverse sampling of 7.5 µm/pixel. Electrical stimulation was incorporated within the first 30 s of the volume scans.

### Capillary velocity analysis

ED-based capillary velocimetry analysis was performed based on repeated 50 A-scans acquired. Details of the velocimetry analysis is demonstrated in our previous publication^26^. Briefly, frequency analyses were firstly conducted using the covariance matrix of grouped A-lines (50 repetitions), with which the eigenvalues and eigenvectors that represent the subsets of the signal markup were calculated. The eigen values and vectors that are due to moving RBCs were isolated via an adaptive regression filter to remove the eigencomponents that represent static tissue. And then the mean frequency (MF) of moving RBC were calculated through first lag-one autocorrelation of the obtained eigenvectors. According to Wang *et al*., the measured MF is linearly related to the mean RBC velocity in single-file passages as verified by a phantom experiment. All MF values in the 3-D space within the scanned tissue bed are used for calculating capillary flow parameters, including mean and spatial distribution of transit time.

### Capillary flow parameter evaluation

All MF values were converted to velocities based on linear function^26^, and the mean of all RBC velocities within 3-D space of each dataset is represented with mean transit velocity (MTV). For MTT and CTTH measurements in the capillary bed, however, transit time of the RBC must be resolved. In doing so, we simply adopted the method in the modeling study^13^ with assumption of a uniform capillary path length *L* = 400 μm to convert velocity to time. After obtaining the RBC transit times from velocities, the values in each 3-D dataset were plotted into a histogram distribution. To validate MTT and CTTH change with the modeled transit time function, where capillary transit time distribution is expressed in gamma function of *τ* modulated by shape and scale parameters of *α* and *β* in Eq. (1)

1 $$h(\tau ;\alpha ,\beta )=\frac{1}{{\beta }^{\alpha }\Gamma (\alpha )}{\tau }^{\alpha -1}{e}^{-\tau /\beta }$$

we fitted our data points on the transit time distribution to a gamma function curve to derive MTT from *αβ* and the standard deviation CTTH from $\sqrt{\alpha }\beta$. These values and their alterations are then compared between rest and stimulation, HLS1 and CTRL, respectively.

### Statistical analyses

All capillary flow parameters are expressed as mean ± std (n = 12). The relative change in the hemodynamic parameters, such as ΔMTV, ΔMTT, and ΔCTTH, from rest to stimulation were statistically tested using Student *t* tests (two-tailed) between HLS1 and CTRL. *P* < 0.05 was considered significant.

### Data availability

All datasets generated and analyzed during the current study are available from the corresponding author on reasonable request

## Results

### Activation at HLS1 upon hindpaw electrical stimulation

**Figure 2:**
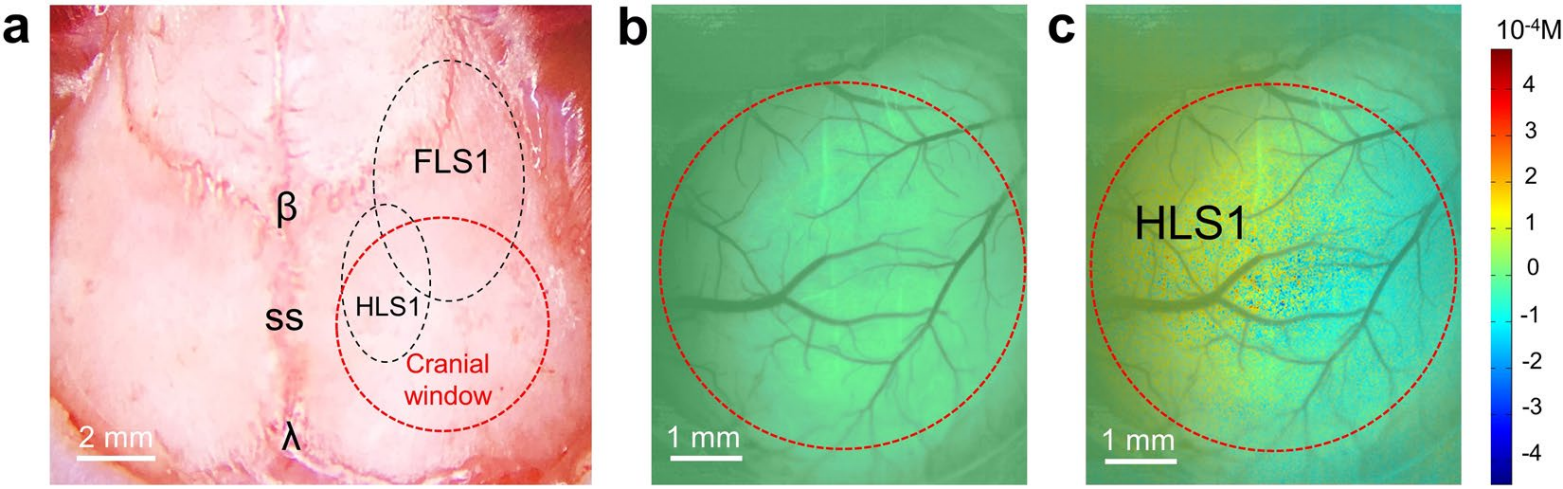
Oxygenation maps revealed by LSCI. (**a**) Light microscopic image giving the relative location of cranial window to the FLS1 and HLS1. Red dashed circle shows the location of cranial window to be created, 1 mm posterior and lateral to bregma. Black dashed regions indicate the approximate locations of HLS1 and FLS1^32^. (**b**) and (**c**) ΔHb during rest and stimulation, respectively, overlaid with arterial angiogram inside the cranial window. Color bar represents Hb concentration differences in μM. The region shown with warmer color in (**c**) corresponds to a higher ΔHb level; thus, indicating oxygen consumption at the HLS1 region during functional activation. β, bregma; λ, lambda; SS, sagittal suture; FLS1, forelimb somatosensory cortex; HLS1, hindlimb somatosensory cortex.

Spatial relationship between the cranial window and mouse primary somatosensory cortex, including both forelimb (FLS1) and hindlimb (HLS1), was identified using the method described in previous study^32^ and demonstrated on the microscopic image of mouse intact skull before cranial window surgery (Fig. 2a). To precisely locate the HLS1, LSCI imaging was performed at the cranial window during rest (Fig. 2b) and hindpaw electrical stimulation (Fig. 2c). Localized increase of ∆Hb were observed during stimulation, indicating an increased utilization of oxygen at the HLS1 (shown as warmer color in Fig. 2c). The oxygenation mapping validated stimulus-evoked activation at HLS1 and provided guidance for the investigation of localized changes in capillary flow pattern in the following experiments.

### OCTA angiogram and velocimetry at HLS1 and CTRL

**Figure 3:**
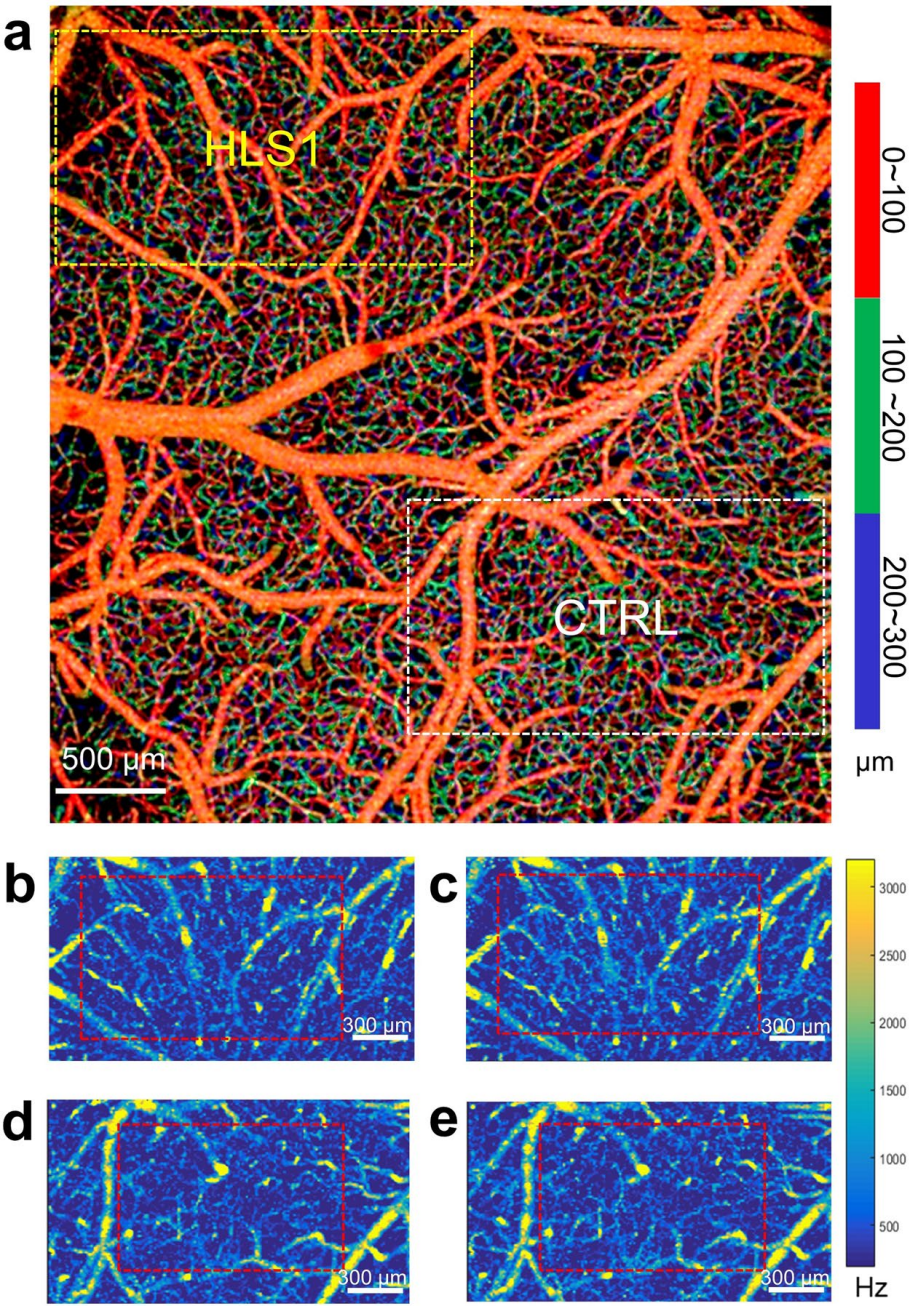
Cerebral angiogram and capillary velocity maps. (**a**) *En face* maximum intensity projection (MIP) of the 3-D OMAG dataset inside the cranial window within 300 µm depth from the cortical surface. Colors represent vessels at depths of 0~100 µm (red), 100~200 µm (green), and 200~300 µm (blue). Dashed squares indicate regions where OCTA velocimetry scans were performed. The yellow square encloses the vascular bed inside activation center (HLS1) as indicated by previous ΔHb maps, and the white square marks a control region (CTRL) further away from the activation center with no noticeable oxygenation consumption change. *En face* average intensity projection (AIP) images of the 3-D MF maps are shown within 300 µm thick slab from the cortical surface for HLS1 during (**b**) rest and (**c**) stimulation, and for CTRL during (**d**) rest and (**e**) stimulation. Color bar represents MF values. The red dashed squares indicate the regions selected for velocity distribution analysis, avoid including large pial arterioles.

Cerebral angiogram inside the cranial window was obtained from x-y *en face* maximum projection (MIP) of the volumetric 3-D OMAG dataset (Fig. 3a). Within 300 µm thick slab from the cortical surface, the depth locations of vessel in axial space (z) are visualized with colors. Red, green, and blue represents vessels from surface pial vessels to deeper capillary vessels, respectively, with each color occupying a 100-µm thick slab as measured from the cortical surface (identified from 3D OCT structural image). Guided with oxygenation mapping obtained from LSCI, a cortical region with increased oxygenation, HLS1, and non-activated region, CTRL, were selected for velocimetry scans during rest and trials of stimulation. MF maps from the 3-D velocimetry scans are displayed with x-y *en face* average intensity projection (AIP) for HLS1 during rest (Fig. 3b) and stimulation (Fig. 3c), and for CTRL during rest (Fig. 3d) and stimulation (Fig. 3e). In these maps, each signal points represent an MF analyzed from dynamic RBC movement, with the MF value (Hz) indicated on the color bar. In each of the four maps, >20,000 MF signals were obtained from the 3-D space within 300 µm thick slab from the cortical surface.

### Capillary transit time distribution change and CTTH reduction at HLS1

**Figure 4:**
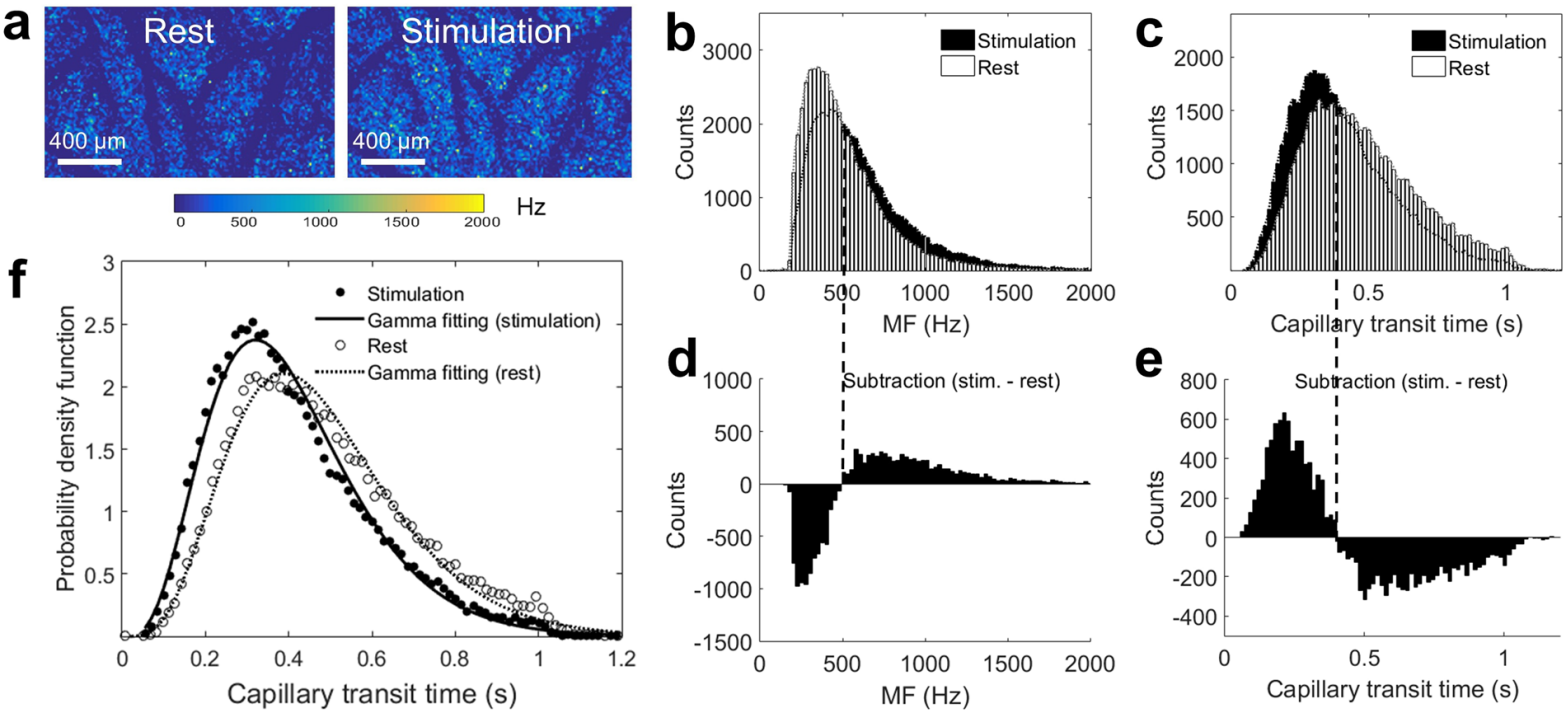
Capillary transit time distribution at HLS1. (**a**) MF maps shown are AIP from 3-D datasets within 300 µm thick slab from the cortical surface after removal of larger surface arterioles (>15 μm) during rest and stimulation. (**b**) Histogram distributions of MFs obtained from rest and stimulation. (**c**) Histogram distribution of capillary transit time during rest and stimulation. (**d**) The differentiation between the histogram functions in (**b**). (**e**) The differentiation between the histogram functions in (**c**). Black dashed lines mark the switch between positive and negative values from differentiations. (**f**) Gamma function fitting for the capillary transit time distribution in (**c**). The coefficient of determination in the fittings are R^2^ = 0.9873 for rest (dashed line) and 0.9811 for stimulation (solid line).

Spatial distributions of MF and transit times were evaluated at HLS1 during rest and upon electrical stimulation. For more accurate evaluation of capillary flow dynamics, additional segmentation was performed to remove MF signals in larger vessels with a diameter of >15 µm, and MF *en face* projection maps after segmentation are shown for rest and stimulation (Fig. 4a). The MF values from these two mapping areas are plotted into a histogram distribution with white bars represent rest and black bars represent stimulation (Fig. 4b). Differentiation of the two distributions were performed (Fig. 4d), indicating that the RBC velocity in most of the capillaries are statistically shifting to slow velocity, with the counts of faster flow velocity becoming less. Capillary transit time were converted from MF-derived velocity, and plotted into histogram distribution as well (Fig. 4c). The differentiation between the rest and stimulation indicates that the transit times are statistically shifting to lower value during stimulation, while the counts of longer transit time become decreased (Fig. 4e). To derive MTT and CTTH values^13^, we fitted two histograms into gamma function curves (Fig. 4f) and calculated the MTT by *αβ* and the CTTH by $\sqrt{\alpha }\beta$. From measuring the relative changes in these quantities at HLS1 from 12 animals, we have observed 9.8% ± 2.2 reduction of MTT and 5.9% ± 1.4 reduction of CTTH from rest to stimulation.

### Capillary transit time distribution at control region

**Figure 5:**
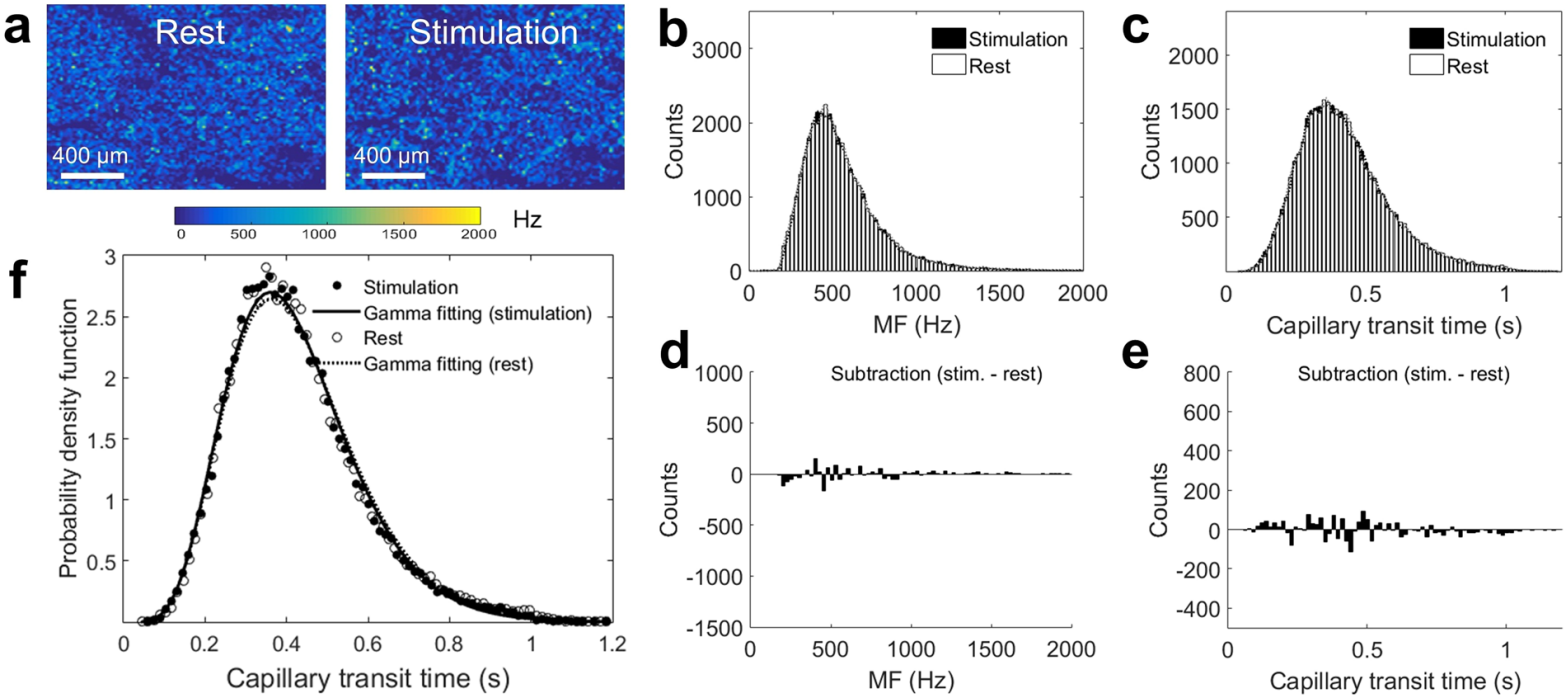
Capillary transit time distribution at control region. (**a**) MF maps at CTRL region are shown for rest and stimulation. (**b**) and (**c**) are histogram distributions of MF and capillary transit time, respectively. The differentiation between the histogram functions from rest to stimulation are shown for (**d**) MF and (**e**) transit time. Differences are not obvious between the probability of rest and stimulation, and no clear switch were identified from negative to positive values. (**f**) Gamma function fitting for the capillary transit time distribution where R^2^ = 0.9796 and 0.9922 for rest and stimulation, respectively.

Capillary flow distribution change at CTRL are also demonstrated. MF *en face* projection maps after segmentation are shown for rest and stimulation (Fig. 5a). From the histogram distribution of MF (Fig. 5b) and transit time (Fig. 5c), the differentiation between rest and stimulation (Fig. 5d,e), as well as the nearly overlapping gamma function curves (Fig. 5f), one can conclude a non-significant change in capillary flow pattern during hindpaw electrical stimulation. Such observation was consistent in the non-activated region from 12 animals.

### Hemodynamic parameters and their relative changes upon stimulation at HLS1 and CTRL

**Figure 6:**
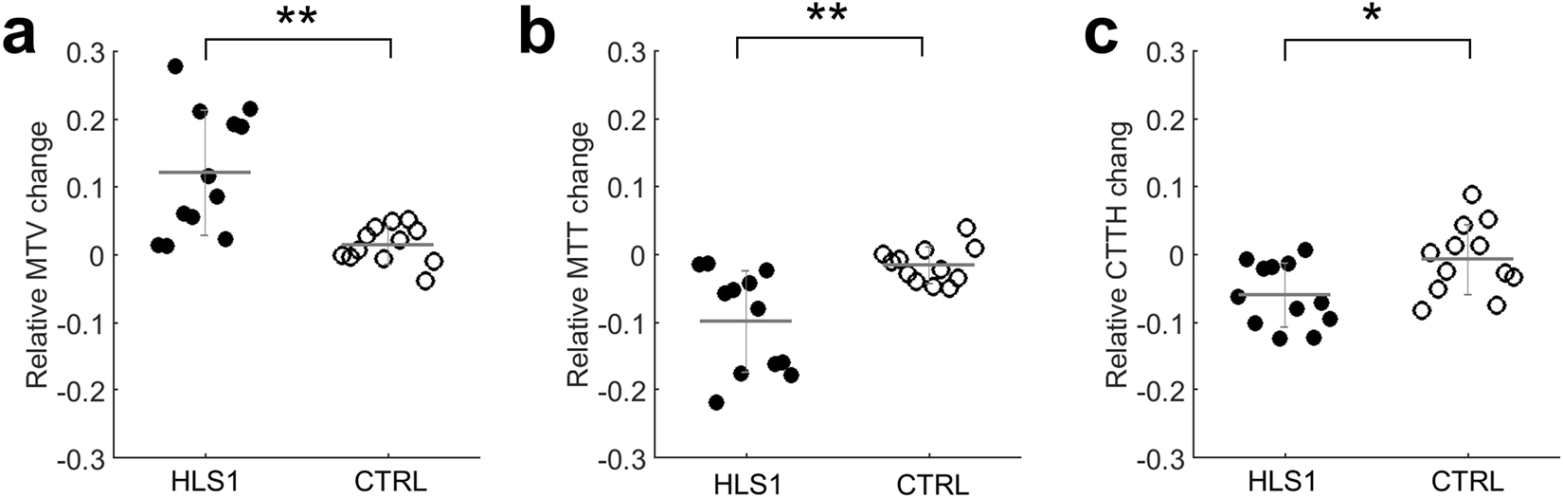
Statistical comparison of capillary parameters between HLS1 and CTRL. Relative change of capillary parameters compared between HLS1 and CTRL for (**a**) ∆MTV, (**b**) ∆MTT, and (**c**) ∆CTTH. *Represents *p* < 0.05 and **represents *p* < 0.01 (*t* test, two-tail).

The relative changes (rest to stimulation) of hemodynamic parameters are compared between HLS1 and CTRL. Significant differences of ∆MTV (*t* test, *p* < 0.01) (Fig. 6a) and ∆MTT (*t* test, *p* < 0.01) (Fig. 6b) were observed between two regions upon electrical stimulation, which indicates a higher transit velocity and lower transit time of the RBC traveling in the capillary bed, regional to HLS1 during hindpaw electrical stimulation. Correspondingly, significant differences of ∆CTTH (*t* test, *p* < 0.05) (Fig. 6c) were revealed between two regions, indicating a local capillary homogenization to HLS1 only, not globally across all cortical regions. Table 1 listed the MTV, MTT, and CTTH measured from both regions at rest and stimulation. Values represent mean ± std from 12 animals.

**Table 1:** Capillary flow parameter measurements at two regions for rest and stimulation (n = 12).

Capillary flow Parameters (Mean ± std)	HLS1		CTRL	
	Rest	Stimulation	Rest	Stimulation
MTV (mm/s)	1.037 ± 0.223	1.146 ± 0.229	1.059 ± 0.212	1.077 ± 0.228
MTT (s)	0.412 ± 0.134	0.367 ± 0.098	0.387 ± 0.110	0.382 ± 0.112
CTTH (s)	0.233 ± 0.079	0.218 ± 0.070	0.229 ± 0.063	0.227 ± 0.059

## Discussion

The current understanding about flow-metabolism coupling is incomplete. During cerebral functional activation, for instance, CBF and glucose metabolism remain coupled as they increase in proportion, whereas oxygen metabolism only increases to a minor degree, so-called uncoupling of CBF and CMRO^2^ that produces BOLD signal in fMRI^6,12^. Reports have speculated about the existence of a potential microcirculatory adjustment in capillary bed where oxygenation occurs. Using accepted diffusion properties of single capillaries, elaborate model shows that it is a basic property of the spatial organization of capillaries that oxygen extraction capacity depends not only on tissue oxygen tension and arterial tone (as quantified by CBV, CBF and MTT), but also to a large extent the distribution of capillary transit times. According to such, the Jespersen&Østergaard model has introduced a crucial physiological effect of CTTH reductions that seemingly counteract the drop in OEF that invariably occurs during functional hyperemia^13^.

Using the transit time parameters introduced in previous framework^13^, we investigated the effect of stimulus-evoked functional activation on microcirculatory hemodynamics in the mouse brain cortex using OCTA velocimetry, based on ED-based frequency analysis of RBC signals within the ensemble of repeated OCT A-scans. This imaging approach enabled >20,000 frequency-derived RBC velocities to be analyzed from each 3-D tissue volume within 75 s. Reported RBC velocity (*v*) distributions were converted to transit time (*τ*) distributions assuming *τ* = *L/v*, where L is the length of the capillary path along which RBC exchanges oxygen with tissue before it converges to draining venules^18,33^. We adopted the value of *L* = 400 μm reported in the literature^18,33^ as a conservative estimate of the RBC travel length in the capillary path to obtain the transit time distribution, upon which the spatial heterogeneity of capillary flow, CTTH, is derived in resting state and during hemodynamic response. In addition, our study employed the state-of-art LSCI to generate oxygenation maps to reveal functional activation with an increase in ΔHb, which indicates relative increase in CMRO^2,29^. This provides evidence of concomitant oxygen metabolism to guide for OCTA scans at designated region and, for the first time, provides the validation of correlation of capillary flow homogenization with localized oxygen consumption. In our experiments, reductions in both MTT and CTTH were seen upon hindpaw electrical stimulation. Such changes were only observed at the cortex corresponding to hindlimb region (HLS1). The differences in hemodynamic response between HLS1 and the non-activated region is statistically significant (two-tail *t* test, ∆MTV *p* < 0.01, ∆CTTH *p* < 0.05). By plugging our observations into the Jespersen&Østergaard model^13^, we were able to confirm the correlation of capillary flow homogenization (CTTH reduction) with functional hyperemia (increased CBF and decreased MTT) during the increased demand of oxygen metabolism (increase in ΔHb) at activated tissue bed.

The *in vivo* observations of reduction in MTT and CTTH also well corroborate the prior findings using bolus tracking with TPM conducted in similar stimulation regime^15^. However, the magnitude of reduction measured in this study, 9.8% ± 2.2 for MTT and 5.9% ± 1.4 for CTTH, is less than the estimated values when using bolus tracking approach, where decreases of 11.3% ± 1.3 and 24.1% ± 1.6 were seen in CTTH and MTT, respectively, during electrical stimulation. This discrepancy may be due to the nature of signals measured. Bolus tracking measures the transport function of plasma dye, and their hemodynamic variables (therefore indicating plasma dynamics), whereas OCTA velocimetry evaluates directly the moving RBCs. Difference between plasma and erythrocyte (RBC) transit times have been previously modeled and estimated (25%)^34^. Thus, MTT and CTTH values cannot be simply compared between two approaches. Before exact relationship of dissociation between plasma and erythrocyte transit time was established, OCTA velocimetry possesses an advantage in estimating RBC transit time distribution for capillary flow parameter measurements.

From the ED-based frequency analysis, we obtained MF representing RBC mean velocity in capillary passages. It was also noted that the temporal fluctuation of the frequency signal within the time interval of 400 µs (50 A-scans), termed bandwidth frequency (BF), could also be obtained from analyses, which represents temporal heterogeneity of RBC in spatial capillary network^26^. Though not utilized in this current study, the distribution and relative change of BF will be investigated in the future. Such useful ultra-microscopic perspectives may uncover the spatiotemporal relationship between capillary flow response and brain oxygenation during functional activation, which may potentially yield additional insight to the flow-metabolism coupling mechanism.

The current method is not without limitations. It was previously reported that the most pronounced changes in RBC velocity and flux were observed beyond depths of 200 µm^15^, corresponding to layer II and III^35^. The capillary velocity measurements in the current study were performed within 300 µm thick slab from the cortical surface that covered the region of significant capillary flow change. However, to avoid biased measurement in deeper cortical layer due to multiple light scattering that causes optical signal attenuation, inner cortex (depths > 300 µm) was not included in the analyses, and capillary flow response beyond such region remains to be elucidated. In this current study, a linear relationship function between MF and capillary velocity was used to differentiate RBC speed information from the complex OCT signals, but the exact correlation considering the size and shape of RBC, hematocrit density has not been fully explored. Neither limitations, however, are thought to affect our comparisons of relative change in capillary parameters between two cortical regions to reveal localized capillary response and CTTH reduction to functional activation. Additionally, we notice that the measurement errors of the ΔMTV and ΔMTT in HLS1 are larger than those in CTRL among 12 animals. It may be necessary to increase the subject number and look into the quantitative criterion of ΔHb in relation to the changes in the magnitude of capillary transit time parameters in the future systemic investigation of microvascular contribution to the brain oxygenation.

The use of isoflurane as anesthetic agent in this functional activation study was justified by previous observations that showed the preservation of neurovascular coupling under isoflurane anesthesia^36,37^. We found isoflurane to be an easy-to-manage anesthetic drug, without negative effects on hemodynamics from accumulation of injectable anesthetic agents. However, vasodilatory effect of isoflurane^38^ on capillary flow parameters in resting and functional activation state remains to be elucidated in awake animals.

## Conclusion

We have investigated the microcirculatory adjustment to functional activation at mouse somatosensory cortex upon hindpaw electrical stimulation. The statistical powered ED-based OCTA analysis on capillary transit times in cerebral tissue beds has revealed a decreased MTT in consistency with functional hyperemia (CBF increase) and CTTH reduction locally to increased ΔHb. The results support the important role of capillary flow homogenization to cerebral tissue oxygenation during functional hyperemia. The high spatiotemporal resolution OCTA capillary velocimetry provides the ability to quantitatively evaluate microcirculatory dynamics in rodent brain *in vivo*, which would ultimately be useful in our improved understanding of neurovascular coupling mechanism. The technique and concept can be potentially applied to investigate CTTH change or dysfunction in normal and pathological conditions with flow-metabolism deficiency.
